# Physicochemical and pharmacological evaluation of carvedilol-eudragit^®^ RS100 electrosprayed nanostructures 

**DOI:** 10.22038/ijbms.2019.34246.8139

**Published:** 2019-05

**Authors:** Sevil Selselehjonban, Alireza Garjani, Karim Osouli-Bostanabad, Ali Tanhaei, Shahram Emami, Khosro Adibkia, Mohammad Barzegar-Jalali

**Affiliations:** 1 Research Center for Pharmaceutical Nanotechnology, Biomedicines Institute, Tabriz University of Medical Sciences, Tabriz, Iran; 2 Students Research Committee, Tabriz University of Medical Sciences, Tabriz, Iran; 3 Department of Pharmacology and Toxicology, Faculty of Pharmacy, Tabriz University of Medical Sciences, Tabriz, Iran; 4 Pharmaceutical Analysis Research Center and Faculty of Pharmacy, Tabriz University of Medical Sciences, Tabriz, Iran

**Keywords:** Carvedilol, Electrospray, Eudragit® RS100, In vivo evaluation Nanobeads, Nanofibers

## Abstract

**Objective(s):**

This study was carried out to boost the pharmacologic influence of carvedilol (CAR) (as a poorly water-soluble drug) by developing CAR-eudragit^®^ RS100 (Eud) nanofibers and nanobeads benefiting an electrospraying approach.

**Materials and Methods:**

CAR-Eud nanoformulations with varying ratios (1:5 and 1:10) at total solution concentrations of 10 %, 15 % and 20 % w/v were formulated.

**Results:**

The solution concentration remarkably impressed the size and morphology of the samples; in which, the nanobeads (mean diameter of 135.83 nm) were formed at low solution concentrations and high concentrations led to nanofibers (mean diameter of 193.45 nm) formation. DSC thermographs and PXRD patterns along with FTIR spectrum precisely showed CAR amorphization and no probable chemical interactions between CAR and Eud in the electrosprayed nanosystems. The *in vitro* release considerations demonstrated that the nanoformulations with the drug: polymer ratios of 1:10 and 1:5 depict rapid dissolution rate compared to the physical mixtures (PMs) and the pure drug. The *in vivo* studies in Wistar male rats suggested that the electrosprayed nanoformulation (1:10; 20 %) reduced the isoproterenol (ISO) induced elevation of heart rate, necrosis and accumulation of neutrophils in the heart tissue more efficient than the pure drug and PM.

**Conclusion:**

Our finding illustrated that the electrospraying as a profitable one-step procedure could be productively benefited to improve the physicochemical features and pharmacologic influences of CAR.

## Introduction

Cardiovascular (CVD) and coronary artery diseases (CAD) such as myocardial infarction (MI) are the first leading cause of the global mortality (31 % of worldwide deaths) based on the World Health Organization (WHO) report (1). Carvedilol (CAR) (an antihypertensive substance with nonselective α_1_ and β blocking behaviors) have been broadly prescribed to treat MI, hypertension, heart failure (HF)/ congestive HF and patients with systolic disfunction after MI (2). CAR the 3^rd ^generation β blocker offers multiple advantages (i.e. cardioprotection, vasodilatation, anti-proliferation, anti-oxidant, anti-arrhythmic and metabolic actions) because of its nitric oxide dependent characteristics. However, CAR is a class II drug based on biopharmaceutical classification system (BCS) with low aqueous solubility and consequently poor oral bioavailability (around 25–35 %) (3). These drawbacks (i.e. low oral bioavailability and poor aqueous solubility) are associated with 40 % of newly discovered active pharmaceutical ingredients (APIs) as reported in literature (4). So, different methods, including cyclodextrin complexation (5, 6), solid dispersion (7), cocrystallization (8), nanoemulsion (9, 10), liquisolid (11) and salt formation (12) have been developed to enhance solubility and consequently the bioavailability of such APIs. Nowadays, drug loaded nanoparticles (NPs) have been considered as the most attractive drug delivery systems (DDSs) with ability of delivering a precise dose of drugs to the action site and enhancing therapeutic effect alongside decreasing patient compliance and drug toxicity (13-16). Furthermore, these controllable and suitable carriers can significantly improve the APIs dissolution rate due to the particle size reduction (Noyes–Whitney equation) which in turn could lead the drug bioavailability enhancement. Various inorganic, organometallic and organic compounds such as polymers, liposomes, lipids, micelles and viruses properly have been used to design applicable API nanoparticles with enhanced functionality. The polymer based NPs (PBNPs) and drug entrapment in these PBNPs are growing interest of new drug formulation systems (17, 18). In comparison with the conventional drug formulations, better delivery control can be achieved using the drug entrapped PBNPs. Many strategies such as an aerosol flow reactor, supercritical fluid (19), solvent evaporation/emulsification (13), template synthesis (20) and electrospraying/electrospinning (20-22) have been advanced to produce the PBNPs. 

Recently, numerous attempts have been made for manufacturing appropriate micro-nanoscale beads or fibers by electrohydrodynamic or electrospraying method to be exploited in DDSs, optoelectronics, microelectronic, tissue engineering, etc. (21-23). This method atomizes a compound of a polymer-drug solution using a high voltage (i.e. 20-30 kV) applied to a syringe (capillary nozzle) (20, 23). The high electrical force and consequently electrostatic charge build-up on the tip of the nozzle cause a shape change in the solution interface. Enhancing the electrical potential leads to increase of the electrostatic forces and as a consequence reduces the surface tension effect on the interface shape at the nozzle tip and the Taylor cone forms once a balance between the two forces is achieved. The cone will break into droplets with smaller sizes if further charge disturbs the cone tip. Flow rate, conductivity, viscosity and surface tension, are the parameters that affect the size of resulted droplets. Normally, fiber formation predominated when the viscoelastic forces overcome the surface tension due to the solution concentration enhancement in electrospraying procedure (23). The electrospray produces a highly charged liquid jet, which moves out towards a grounded screen/counter electrode in the high voltage electrical field. During the jet flying, solvent evaporates and drug encapsulated nanofibers or nanobeads are developed based the polymer-drug solution viscosity (23). By applying this one-step method at ambient pressure and temperature, nanosystems with high throughput, negligible drug loss, high loading capacity and uniform drug dispersion inside the polymeric matrix can be achieved. Ability of controlling the quality of sprayed nanobeads and nanofibers; capability of spraying the most of polymers without changing the basic setup of the process; cost effectiveness and ease of operation are the other valuable aspects of this method (24). Some drugs, such as propranolol hydrochloride (23), insulin (25), triamcinolone acetonide (26), paclitaxel (27), ibuprofen (28), azithromycin (29), naproxen (30) and methylprednisolone acetate (31) have been successfully processed using this method.

Eudragit^®^ RS100 (Eud) is a hydrophilic water-insoluble copolymer of poly (ethylacrylate, methyl-methacrylate and chlorotrimethyl-ammonioethyl methacrylate) containing quaternary ammonium groups (4.5- 6.8 %). Some exclusive properties such as good stability, no toxicity, swelling ability in aqueous media and high permeability make it a suitable candidate for drug loading purposes which represents the good material for the drug dispersion. These characteristics may subsequently maximize the cellular uptake of drug–polymer complex. Eud has been previously applied for delivery, as well as increasing the bioavailability of several drugs (13, 23, 26, 29, 31). Furthermore, our previous studies showed that Eud has good characteristics and processability for applying in electrospray technique (13, 23, 26, 29, 31). Although there are some reports regarding preparation of CAR electrospuned fibers using various polymers such as eudragit^®^ L 100-55 (poly (methacylic acid-co-methyl methacrylate)), eudragit^®^ E (poly (butyl methacrylate-co-(2 demethylaminoeethyl) methacrylate co methyl methacrylate) (32), eudragit^®^ EPO (poly (butyl methacrylate co (2 dimethylaminoethyl) methacrylate co methyl methacrylate) 1:2:1) (32, 33), polycaprolactone, polyvinylpyrrolidone K90 (32) and copolymer of vinylpyrrolidone-vinyl acetate (kollidon^®^ VA64) (34), despite that, to the best of our knowledge, there is no report regarding the CAR- Eudragit^®^ RS100 electrosprayed nanosystems preparation and their *in vivo* characterization.

In this study, we focused on the CAR-Eud nanosystems development in order to improve the physicochemical characteristics as well as the pharmacological effect of CAR. To this end, CAR- Eud nanobeads and nanofibers were formulated using the electrospraying method with different drug to polymer ratios at various solution concentrations. The prepared nanosystems were assessed for the morphological and physicochemical behaviors. Furthermore, we made special focus on pharmacological effects of the prepared nanosystems in Wistar male rats.

## Materials and Methods

***Materials***

Eudragit^®^ RS100, Carvedilol and isoproterenol were obtained from Degussa (Darmstadat, Germany), Salehanchemi (Tehran, Iran) and Sigma-Aldrich (USA), respectively. Sodium hydroxide, ethanol and potassium phosphate monobasic were purchased from Merck (Germany). All other chemical materials were analytical grade.

***Electrospraying samples preparation***

A custom-designed electrospraying apparatus (Fanavaran Nano-Meghyas, Tehran, Iran) was operated to formulate CAR-Eud samples. Briefly, CAR-Eud solutions with 1:5 and 1:10 drug: polymer ratios were developed by co-dissolving CAR and Eud in ethanol at ambient condition. The drug: polymer solution total concentrations were regulated to be 10, 15 and 20 % (w/v).

The liquid stream (jet) of formulated solutions was shaped by utilizing a voltage of 25 kV connected to the syringe tip (gauge 29) attached to a ring shaped capillary polyethylene vessel with internal diameter of 0.1 mm. The processed solutions were streamed upon a grounded polytetrafluoroethylene coated aluminum, as a collector screen to produce CAR-Eud systems. The injection rate and distance between the grounded screen and nozzle tip were fixed at 5 ml/hr and 10 cm, respectively. In this study a piece of an inert and rigid thermoset polymer with a sharp edge was used to collect the sprayed formulations from the surface of the collector screen in order to do further studies. Furthermore, the tumbling bottle technique was applied to prepare the corresponding physical mixtures, where the tumbling time was set to be one hour in a 100 ml bottle to attain homogeneous PMs.

***Field emission scanning electron microscopy (FE-SEM)***

The field emission scanning electron microscope (MIRA3, Tescan Co, Brno, Czech) acting at 20 kV was benefited to assay the processed specimens morphology. Prior to investigate, a thin gold film was used to coat the electrosprayed formulations (about 150 Å in thickness) using gold sputtering machine (Emitech K550, Kent, UK). The average diameters of electrosprayed samples were estimated directly from FE-SEM images by calculating the samples diameters at above 50 points applying Digimizer image analysis software. The assessed diameters were illustrated as ‘‘mean Feret diameter±standard deviation’’. A particle size measurement along a particular direction is called the Feret/Feret’s diameter. Generally, it can be described as the space between two parallel tangential lines that perpendicularly limits the particle to that direction. This method is applied to measure particle sizes in microscopy, where a 3-dimensional particle is projected on a 2-dimensional plane (7, 32, 35). 

***Differential scanning calorimetry (DSC)***

The thermograms and thermal behaviors of pure CAR, Eud, physical mixture (PM) and electrosprayed formulations were studied conducting DSC 60 (Shimadzu, Kyoto, Japan). Briefly, at first 5 mg of each processed sample was meticulously weighed and placed in sealed aluminum pans, then the thermal attitudes were investigated at a scan rate of 20 ^°^C/min (25-220 ^°^C) and analyzed by TA60 software. The indium and aluminum oxide powders were served as standard and reference models, respectively. 

***Fourier transform infrared spectroscopy (FTIR)***

The FTIR spectrophotometer (Shimadzu 43000, Kyoto, Japan) was utilized to justify the chemically probable interactions of drug-polymer. Briefly, a compact disc of pure CAR, Eud, CAR-Eud PM and their electrosprayed specimens were manufactured by KBr disk approach and assessed at scanning range of 4000-400 cm^-1^ with average spectra of 32 scans at a resolution of 2 cm^-1^.

***Powder X-ray diffraction (PXRD)***

PXRD patterns of pure CAR, Eud, PM and electrosprayed samples were achieved employing X-ray diffractometer D5000 (Siemens, Munich, Germany) at 2θ angle range, scanning rate and step size of 10^o^-40^o^, 0.6 ^o^/min and 0.02^o^, respectively by K_α_ radiation of Cu (λ=1.5405 Å) at 40 kV, 30 mA.

***In vitro drug release***

The dissolution characteristics of pure CAR, PM and electrosprayed samples were studied using USP apparatus II (paddle method). Briefly, samples equivalent to 20 mg of CAR under rotational agitating (50 rpm) were situated in the container with 300 ml of phosphate buffer (pH 6.8) at 37±0.2 ^°^C. At predetermined intervals, 3 ml of the treated solutions was removed and replaced with the same amount of fresh buffer in order to retain an unvarying volume. The cellulose acetate membrane (pore size 20 nm, Whatman, Kent, UK) was utilized to filter the carried away solution. UV spectrophotometer (Shimadzu, Kyoto, Japan) at a wavelength of 285 nm was conducted to estimate the drug cumulative release graphs. The average values of three assessments were used.

**Figure 1:**
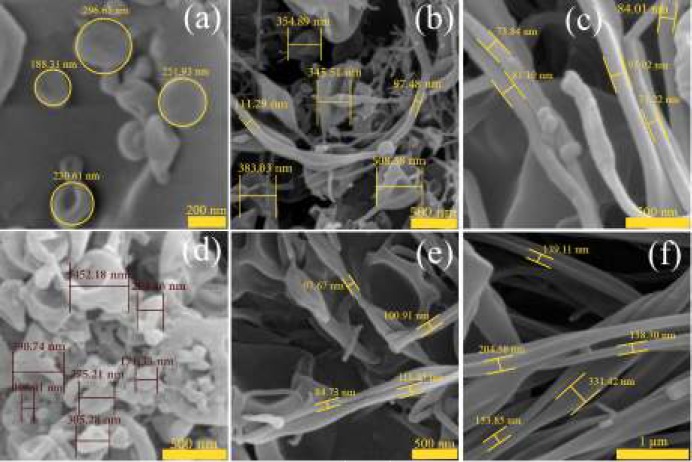
Field emission scanning electron microscopy images of the carvedilol-eudragit^®^ RS100 electrosprayed nanosystems with (a) drug: polymer ratio of 1:5 at the total solution concentration of 10 % w/v (Magnification: ×100 k), (b) 1:5-15 % w/v (×50 k), (c) 1:5-20 % w/v (×70 k), (d) 1:10-10 % w/v (×70 k), (e) 1:10-15 % w/v (×50 k) and (f) 1:10-20 % w/v (×40 k)

**Figure 2:**
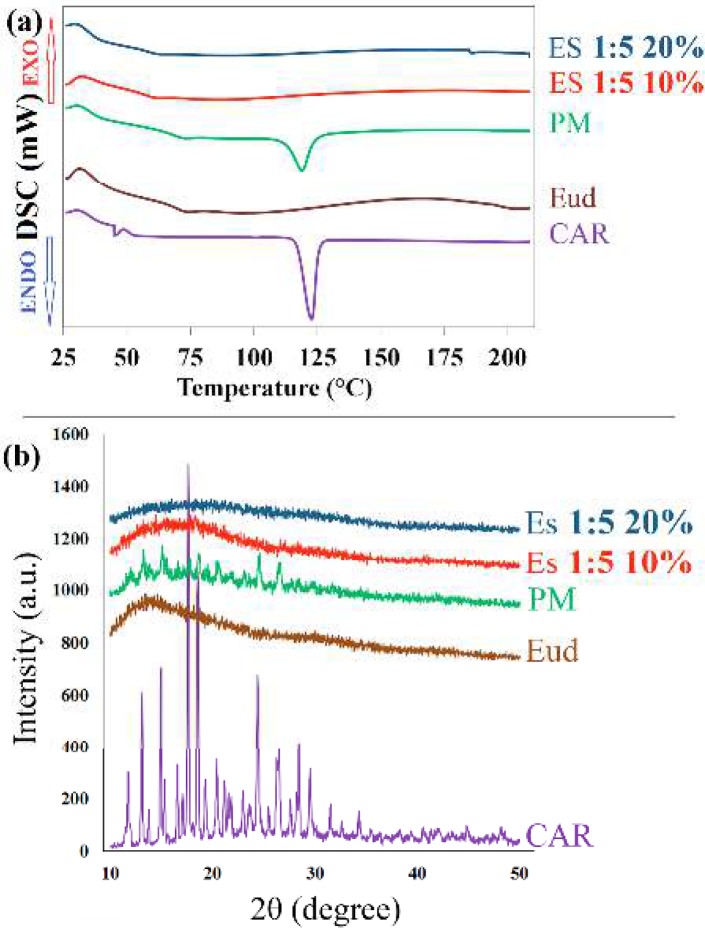
(a) Differential scanning calorimetry thermograms and (b) powder x-ray diffraction patterns of the pure carvedilol (CAR), eudragit^®^ RS100 (Eud), physical mixture (PM) and electrosprayed nanosystems (ES) with the drug: polymer ratio of 1:5 at total solution concentrations of 10% and 20% (w/v)

**Figure 3:**
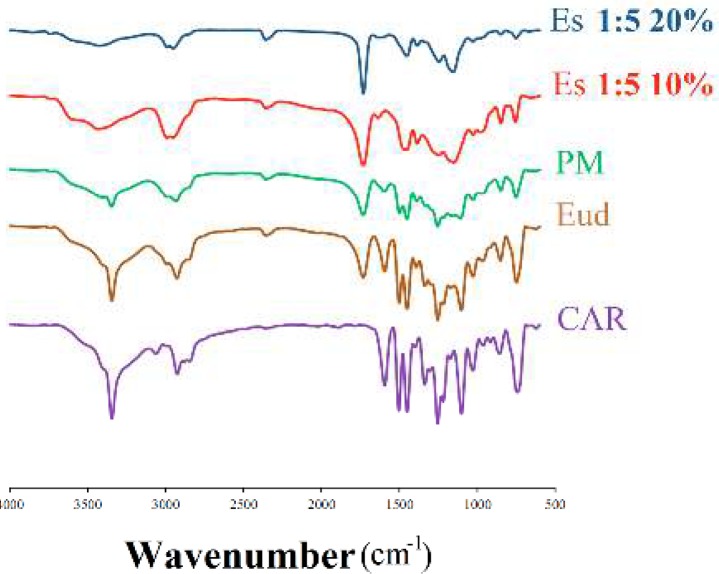
Fourier-transform infrared spectroscopy curves of the pure carvedilol (CAR), eudragit^®^ RS100 (Eud), physical mixture (PM) and electrosprayed nanosystems (ES) with the drug: polymer ratio of 1:5 at total solution concentrations of 10% and 20% (w/v)

**Figure 4:**
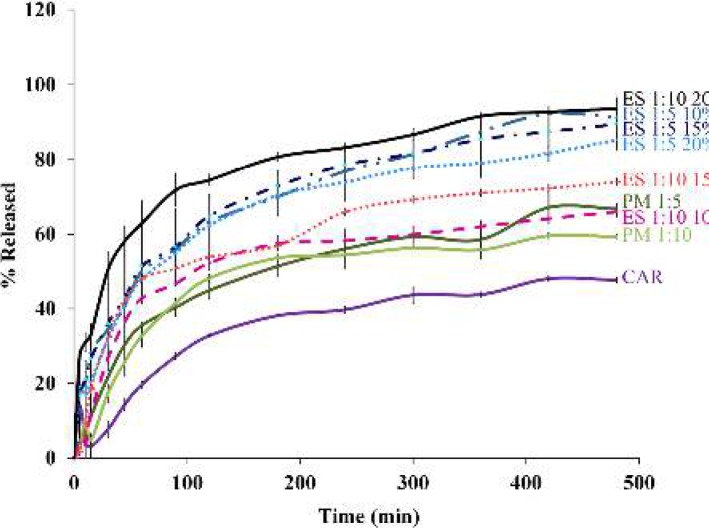
Dissolution profiles of the pure carvedilol (CAR), physical mixtures (PM) with drug: polymer ratios of 1:5 and 1:10, and electrosprayed nanosystems (ES) with the drug: polymer ratios of 1:5 and 1:10 at total solution concentrations of 10 %, 15 % and 20 % (w/v)

**Figure 5:**
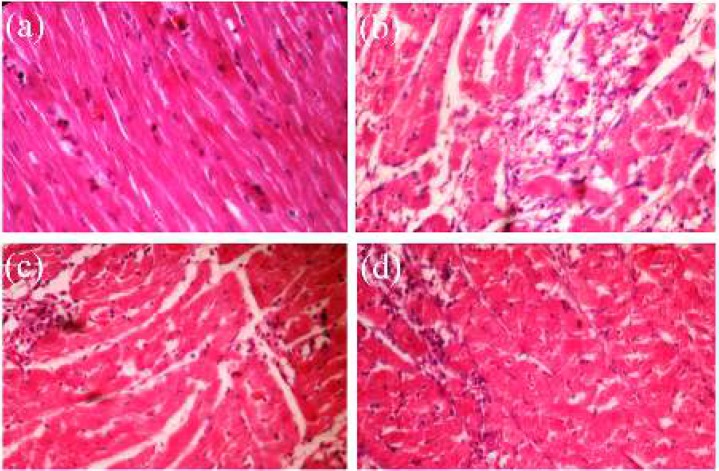
Microscopic images of cross sections of rat heart apexes (a) control group (saline (sal)+sal), (b) isoproterenol injected (ISO+sal), (c) ISO+ physical mixture of the carvedilol- eudragit^®^ RS100 (drug: polymer ratio of 1:10) (ISO+PM) and (d) ISO+ES (electrosprayed nanosystems (drug: polymer ratio of 1:10 at total concentration of 20 % w/v)) (40X)

**Table I:** *Summery of the *in vivo* procedure on 6 study groups consisting of 5 rats each.* *Sterile saline (Sal), subcutaneous injection (S.C), isoproterenol (ISO), physical mixture (PM) and electrosprayed formulation (Es)*

Study Groups	Day I	Day II	Day III	Day IV	Day V	Day VI
I	S.C of Sal	S.C of Sal	Sal	Sal	Sal	Sal
II	S.C of ISO	S.C of ISO	Sal	Sal	Sal	Sal
III	S.C of Sal	S.C of Sal	Sal+PM	Sal+PM	Sal+PM	Sal+PM
IV	S.C of Sal	S.C of Sal	Sal+Es	Sal+Es	Sal+Es	Sal+Es
V	S.C of ISO	S.C of ISO	PM	PM	PM	PM
VI	S.C of ISO	S.C of ISO	Es	Es	Es	Es

**Table 2:** *Calculated amounts of the t*_25%_ *, Q*_15min_ *and Q*_30min_ *for pure carvedilol (CAR), physical mixtures (PM) with drug: polymer ratios of 1:5 and 1:10, and electrosprayed nanosystems (ES) with the drug: polymer ratios of 1:5 and 1:10 at total solution concentrations of 10 %, 15 % and 20 % (w/v)*

**sample**	**t**_25% _ **(min)**	**Q**_15 min _ **(%)**	**Q**_30 min (%)_
CAR	85	3.0 ± 2.9	7.6 ± 2.3
PM 1:5	36	11.0 ± 0.4	21.9 ± 0.6
PM 1:10	44	24.0 ± 3.4	17.5 ± 2.6
ES 1:5 10%	13	28.4 ± 3.1	34.5 ± 2.7
ES 1:5 15%	13	26.5 ± 3.9	36.1 ± 1.1
ES 1:5 20%	18	23.5 ± 1.5	34.7 ± 1.3
ES 1:10 10%	28	11.8 ± 1.0	26.6 ± 1.6
ES 1:10 15%	21	18.5 ± 5.4	32.5 ± 5.0
ES 1:10 20%	4	33.3± 2.6	50.3 ± 4.9

**Table 3:** Average values of the recorded heart rate (HR), PR interval (PR), QRS complex (QRS), QT interval (QT), P amplitude, R amplitude and ST height of the experimented rats. (n=5)

Group	HR (beats/min)	PR (msec)	QRS (msec)	QT (msec)	P amplitude (µv)	R amplitude (µv)	ST height (µv)
**CON** (sal+sal)	237.6±4.8	55.8±1.9	21.4±3.1	58.1±9.4	33.9±5.1	334.0±51.2	106.4±24.0
**ISO** (ISO+sal)	282.6±27.8	48.9±4.0	21.5±2.7	57.1±10.6	56.1±18.8	294.6±21.5	66.1±32.5
**ISO+PM**	239.8±8.9	51.2±2.5	25.1±4.2	47.4±5.4	30.0±8.2	409.8±103.7	17.4±5.54
**CON+PM**	224.2±14.5	53.4±3.3	17.4±2.9	65.5±10.5	30.6±8.8	319.8±39.9	38.6±17.7
**CON+ES** (sal+ES)	231.8±7.2	45.5±3.2	15.2±1.0	47.4±6.1	16.8±3.3	156.2±39.5	63.6±18.2
**ISO+ES**	188.8±10.6*	61.4±2.7*	23.8±6.5	59.9±10.7	28.3±6.0*	229.0±69.9	25.8±7.3*

* Statistically significant (p<0.05), CON: Control group, ISO: Isoproterenol, PM: Physical mixture (drug: polymer ratio of 1:10), ES: Electrosprayed sample with the drug: polymer ratios of 1:10 at total solution concentration of 20 % (w/v). The data were reported average ± SEM (standard error of mean).

***Animal study***

*Animals*

A total of 30 male Wistar rats (250-300 g) was supplied by Animal Center Laboratory, Tabriz University of Medical Sciences, Iran. Animals were housed under specific conditions of 12-12 hour light to dark cycle in an air conditioned room at 20±2 ^°^C with a relative humidity of 50±10%. Food (UAR, Villemoissonsur Orge, France) and water were supplied *ad libitum*. All animal procedures were performed according to the ‘Guide for the Care and Use of Laboratory Animals’ of the research center for Laboratory Animal of Tabriz University of Medical Sciences which is in accordance with the National Institutes of Health guidelines (revised 2011) and was approved by the local authorities of Animal Ethics Committees (AEC reference number: IR.TBZMED.REC.1395.1337).

*In vivo procedure*

Healthy adult rats were randomly allocated into 6 groups consisting of 5 rats each. Rat in all groups were gavaged orally either saline or formulations for six consecutive days. Animals in group I (Control (Sal+Sal)) were injected sterile saline (0.5 ml) subcutaneously (SC) for two days and then were gavaged 0.5 ml sterile saline solution for the last four days. Rats in group II (ISO+Sal) were injected isoproterenol (ISO) (100 mg/kg; SC) for two days with an interval of 24 hours and subsequently were gavaged 0.5 ml saline the same as the group I for last four consecutive days. Animals in group III (Sal +PM) were received SC injection of sterile saline in two starting days and then were given orally CAR-Eud physical mixture (2 mg/kg; CAR) in the last four days. Rats in groups IV (Sal+Es) were injected sterile saline (0.5 ml, SC) for two days plus were gavaged CAR-Eud electrosprayed formulations (drug: polymer ratio of 1:10 and total solution concentration of 20 %) for the last 4 days. Rats in groups V (ISO+PM) were injected ISO (100 mg/kg) for two consecutive days and were gavaged PM the same as the group III. Rats in groups VI (ISO+Es) were received SC injection of ISO (100 mg/kg) for two days and then were given CAR– Eud electrosprayed formulations orally the same as the group IV. 

On the sixth day, 2 hours after the last gavage the animals were anesthetized by IP injection of ketamin (400 mg/kg) plus 40 mg/kg xylazin. Then, when rats, no longer responded to external stimuli the standard limb lead II electrocardiogram (ECG) was recorded using POWERLAB system (AD instruments, Australia) for evaluating the heart rate (HR), ST-Segment and R wave amplitude. Table 1 summarizes the *in vivo* procedure of the current study.

In the current study, all the gavaged samples were prepared freshly and immediately before administration using mild ultrasonic vibration for only three seconds that resulted in a very homogenous dispersion of the electrosprayed sample in sterile saline solution. As mentioned previously, Eud is a hydrophilic water-insoluble copolymer that its hydrophilicity helps achieving a homogenous dispersion of the electrosprayed formulation.

*Histopathological evaluation*

In order to carry out the histopathological evaluation, the apex of the dissected hearts was separated and kept at 10 % buffered formalin. Afterwards, the fixed parts were embedded in paraffin, sectioned (5-6 μm thick) and stained by hematoxylin and eosin for regular tissue assessing. Histopathological evaluations, i.e. neutrophils, myocyte necrosis, and edema were accomplished in the blinded way under the BX50 light microscope (Olympus, Tokyo, Japan). The degree of histopathological changes for each of the layers was scaled from 1 to 4: (1) low, (2) mild, (3) moderate and (4) severe injury. 

***Statistical analysis***

One way ANOVA, which followed by a Student–Newman–Keuls post-test was applied to compare the groups. Statistical analysis was implemented using Sigmaplot V12 and any variations among the groups were assumed significant at *P<*0.05 levels. The *in vivo* data were asserted as mean±SEM and attained from five experimented rats.

## Results

***Morphological evaluation of electrosprayed samples***

Figure 1 shows the morphology and size of CAR-Eud electrosprayed systems. As it is clear from the FE-SEM results, the nanobeads in concave shape were formed at lower solution concentrations, so that the average particles size were 237.66±54.17 nm and 369.97 ±108.01 nm for the nanobeads with drug: polymer ratios of 1:5 and 1:10 (total solution concentration of 10% w/v), respectively (Figure 1A, D). Enhancing the solution concentration led to formation of the nanofibers with smooth surfaces at both 1:5 and 1:10 drug: polymer ratios (Figure 1C, E, F). The corresponding average diameters were 80.64±10.90 nm (1:10; 15 % (w/v)), 98.64 ±13.26 nm (1:5; 20 % (w/v)) and 193.45 ±96.57 nm (1:10; 20 % (w/v)). It is worth to note that, the electrosprayed sample with drug: polymer ratio of 1:5 with a total solution concentration of 15 % (w/v) (i.e. a moderate concentration) (Figure 1B) resulted a blend of nanobeads (512.99 ±85.91 nm) and nanofibers (104.24 ±6.76 nm). 

***Differential scanning calorimetry***

The thermal behavior of pure CAR, Eud, PM and electrosprayed nanosystems were examined by DSC (Figure 2A). A sharp endothermic peak at 117 ^°^C was related to the melting point of CAR (33), where Eud showed an amorphous attitude (glass transition temperature of 58.44 ^°^C) (23, 26). The melting peak of CAR was not detected in the electrosprayed nanosystems, suggesting occurrence of one or more of the following events: the drug amorphization; heat induced interaction between CAR and Eud; and the drug solubilization in the polymer. Moreover, the PM of CAR and Eud showed the endothermic peak of the drug with a diminished intensity and a slight shift due to the solubilization of CAR in melted Eud and/or dilution effect of Eud and/or heat induced interaction between CAR and Eud (8, 23). 

***Powder X-ray diffraction (PXRD) evaluation***

The crystallinity of the pure drug, Eud, PM and electrosprayed samples were identified using X-ray diffractometer (Figure 2B). The sharp, distinctive diffraction peaks at 2θ angles of 12.8°, 15.62°, 17.46°, 18.56°, 20.1°, 24.3° and 26.2° were demonstrated the crystalline characteristics of pure CAR (32, 34, 36). The absence of any characteristic peaks in the PXRD curve of Eud revealed its amorphous behavior. The PXRD pattern of PM showed the characteristic peaks of CAR with a reduced intensity due to the possible dilution effect of Eud (37). Although, CAR preserved its crystalline structure in the PM; however, no distinctive diffraction peak was indicated in the PXRD patterns of CAR-Eud electrosprayed nanosystems, suggesting CAR transformation to an amorphous form during the preparation process. 

***Fourier transform infrared spectroscopy***

The FTIR spectrophotometer was applied to detect the possibility of any drug-polymer chemical interactions in the solid state (Figure 3). The FTIR spectrum of CAR displayed distinctive peaks at 3344.66 cm^-1^ (N-H and O-H stretching peaks combined together), 2924.86 cm^-1 ^(C-H stretching), 2840.56 cm^-1 ^(C-O stretching), 1594.90 cm^-1^ (N-H bending vibrations), 1255.39 cm^-1^ (C-O stretching and O-H bending vibrations) and 1029.54 cm^-1 ^(symmetric C-O-C stretching) (33, 36, 38). Moreover, FTIR spectra of pure Eud indicated the peaks at 2991.35 cm^-1 ^(CH aliphatic stretching) and 1732.64 cm^-1 ^(-C=O stretching) (23, 26).

The PM FTIR spectra clearly revealed the drug and polymer characteristic absorption bands, demonstrating presence of CAR and Eud. Additionally, CAR and Eud typical bands were detectable in the FTIR spectrum of the electrosprayed formulations, illustrating the retention of CAR chemical identity. However, CAR peaks intensity was decreased or the peaks were broadened in the prepared formulations, probably because of the crystallinity loss or the dilution effect of the polymer and physical entrapment of the drug within the polymer matrix especially at high solution concentrations. These findings are in good conformity with DSC and PXRD results and other relevant reports (29, 31, 38). 

***In vitro dissolution study***

The drug release profiles of pure CAR, PMs and electrosprayed nanosystems were assessed using USP apparatus II (paddle method) (Figure 4). The effect of electrospraying procedure and the polymer ratio on the drug release behavior were assessed by calculating t_25%_ (the time needed for 25% of the drug release), Q_15min_ and Q_30min_ values (relevant percent of the dissolved drug during 15 and 30 min, respectively) are illustrated in Table 2. 

Considering the t_25%_, Q_15min_ and Q_30min_ values at a specified pH, it can be concluded that the electrosprayed nanosystems had meaningfully faster drug release rate than the PMs and pure drug. So that, Q_15min_= 33.3 % and Q_30min_=50.3 % were determined for the nanoformulations with the drug: polymer ratios of 1:10 at total solution concentration of 20 %, while the corresponding values were estimated to be 3.0 % and 7.6 % for the crystalline CAR.

The dissolution curves (Figure 4) depicted a relatively biphasic release pattern; so that the initial rapid release within the first hours followed by the slow release. Drug encapsulation on the superficial layers of the particles as well as the high specific surface area due to the smaller size of the nanobeads and nanofibers are two phenomena which could explain the rapid release; while, the diffusion and dissolution of the drug from inner layers elucidated plateau phase (23, 29). Altogether, electrosprayed formulations showed enhanced drug release compared to the pure drug and PMs, but it was demanding to explain the mechanism of drug release and randomness of release patterns. Hence, scrutinizing the exact reason of the observed release behaviors needs to conduct further studies. However, by considering the aim of this study, that was improving the pharmacologic influence of carvedilol (as a poorly water-soluble drug), the fastest release rate of the electrosprayed sample with the drug: polymer ratio of 1:10 at the total solution concentration of 20 % (*w/v*) made it an appropriate system for the *in vivo* delivery of CAR.

***In vivo study***

In the present study, maximum effect on heart rate was induced two hours after the last injection of ISO. *In vivo* effectiveness of the CAR-Eud electrosprayed nanosystems were compared with that of the PM and pure CAR by assessing their effects on ECG parameters, incidence of the necrosis and edematous in the heart tissue of the rats. It is mentioned that the hypotension is a pleiotropic effect of carvedilol because of the relative blockage of vascular α receptors (39, 40), but there is not clear and measurable (at least in the experiential model of this study) as well as an immediate action of the drug. Carvedilol mainly is a beta blocker and its original and real pharmacological effects appear on the heart. Therefore, the ECG parameters were evaluated in this study. Table 3 summarizes the data of recorded ECG parameters in the different rat groups. 

As shown in Table 3, the heart rate was increased from (238±5) in normal control rats (sal+sal) to (283±28 beats/min) in the group injected ISO (ISO+sal). Administration of CAR–Eud electrosprayed nanoformulations (drug: polymer ratios of 1:10 at the total concentration of 20 % *w/v* (2 mg/kg; CAR)) (group VI (ISO+ES)) caused a sharp decrease in the heart rates of the ISO injected rats (224±15 vs 283±28; *P<*0.05). Compared to ISO group (ISO+sal) all CAR formulations reduced the heart rate; however, the reduction was not attained a significant level in some groups (except the group VI (ISO+Es)). Additionally, the PR interval (also assigned as the PQ interval, which indicates the depolarization propagation from the atrium to the heart ventricles) significantly was increased in the rats treated with CAR–Eud electrosprayed nanoformulations (61.4±2.7 msec) compared with rats received ISO alone (48.9±4.0 msec; *P<*0.05). Besides, the P amplitude as an index of arterial electrophysical activity significantly was reduced by CAR-Eud nanosystems administration and more interestingly in comparison with ISO group, the ST height (a vital parameter for the diagnosis of myocardial infarction and myocardial ischemia) was also reduced by 40% at the CAR–Eud electrosprayed nanoformulations treated group (group VI (ISO+ES)). These findings are in good agreement with the results of the histopathological evaluation (Figure 5).

The incidence of necrosis and accumulation of neutrophils in the heart tissue of experimented rats were evaluated by scrutinizing the cross section microscopic images of the apex part (Figure 5). The normal control group exhibited regular arrangement of the myocardial fibers with clear striations. In addition, there was no obvious degeneration, necrosis in this group. The histological cross sections of ISO treated cardiac tissue indicated a severe grade of necrosis at cardiomyocytes as well as an increase in the edematous intercellular space after hematoxylin and eosin staining (Figure 5B). 

It was recognized that treatment with CAR (Figure 5C, 5d), especially the electrosprayed nanosystems (Figure 5d), significantly reduced ISO induced necrosis and degeneration (*P<*0.05). In the other word, histological necrosis and edema scores of the both groups treated with the CAR-Eud PM and CAR-Eud electrosprayed nanosystems (1:10; 20 % *w/v*) were reduced from the high pathological changes situation (3.35±0.31, ISO group) to moderate state (2.42±0.33 and 2.00±0.41, respectively). Consequently, both treated groups were protected against ISO-induced necrosis and edema, where the protection effect of electrosprayed nanoformulation was superior to the PM.

## Discussion

Drug delivery mechanisms and drug effectiveness are tremendously affected by the morphological characteristics and size distribution of the drug particles. Besides, the polymer concentration, its nature, chains intermolecular interlocking, diffusion rate, solvent properties and its coulomb forces, solvent evaporation rate as well as several working parameters such as the prepared solution feeding rate, operational voltage and distance of the grounded surface and nozzle are the vital factors can play significant role in the morphological characteristics of electrosprayed samples (23, 41, 42). In the current study, all the above mentioned parameters were kept fixed excluding the ratio of the drug: polymer and the total concentration of formulated solutions. 

The results revealed that the solutions with low concentrations (i.e. 10 % (w/v) at both drug: polymer ratios of 1:5 and 1:10) initiated the beads formation due to the high surface tension of these formulations (26, 31). In other words, high surface tension of the solutions at the formulations with low concentration of the polymer led to the liquid jet dispersion to separate droplets; in contrast, the viscoelastic forces of the solutions at the formulations with high concentration of the polymer resulted in the fiber formation. Additionally, development of the larger nanobeads by enhancing the drug: polymer ratio could be related to electrical conductivity reduction of the prepared solution at the high polymer ratios (23, 29, 42).

As mentioned previously, many novel drug products have poor liquid solubility impeding their pharmaceutical functionality after administering. One strategy to enhance solution concentrations upon drug administration is to raise the specific surface area by reducing the drug particle size. Since particles with smaller size have a much higher surface to volume ratio, an augmentation in the dissolution rate (the dissolved amount of the drug per unit time) is anticipated at the equal driving energy for dissolution. The solubility enhancement might be achieved as well, since based on the Ostwald−Freundlich equation (43, 44), particles with smaller size showed increased solubility as witnessed in *in vitro* dissolution studies (Table 2, Figure 4).

Three primary values (i.e. lattice force of a crystalline structure, cavitation and solvation forces) might control a drug solubility in a solution. Generally, the lattice energy of the crystalline structure which should be overcome to solubilize the drug is superior than the solvation and cavitation forces (44). In this regard, developing amorphous formulations benefiting electrospraying procedure could reduce the lattice energy by destroying the crystalline structure of the drug in the delivery stage leading to the augmented dissolution behavior of the formulations. These results have good consistency with DSC findings and previously published studies (23, 26, 29, 31).

The amorphization of CAR within the electrospray procedure could be explained because of the large surface formation of the jet stream during the process that cause rapid evaporation of the solvent and lead to the polymer matrix quick congealing as well as decreasing mobility of the drug molecules. This short drying period and CAR restrained mobility could hinder crystallization of the drug molecules. Furthermore, it has been reported that interactions between the polymer and drug can restrain the drug transformation to the crystalline form, immediately (36, 45).

The observed enhanced drug dissolution rate of the electrosprayed nanosystems could be attributed to: a) Drug amorphization during the fabrication process (as witnessed by DSC and PXRD data), and thoroughly dispersion of CAR in the Eud matrix, so there is no need to extra forces for overcoming the crystal lattice energy. b) Elevation of the specific surface area owing to the particle size reduction, thus the drug dissolution rate is improved according to the Noyes-Whitney equation. c) Decrease of the diffusion layer thickness around the formulated drug as a result of size reduction (36, 42). Furthermore, in the colloidal systems, the saturation solubility is increased by decreasing the particles size which in turns causes the dissolution rate augmentation (44).

Heart failure is one of the major health problems worldwide, caused by the range of heart damages, including pericardium, myocardial and endocardium disorders. The majority of patients with the heart failure suffer from myocardial incompatibility. Anti-adrenergic drugs that block the β adrenergic receptors, are effective candidates for managing this failure (1, 2, 46). CAR is a non-selective β-blocker frequently used to treat the heart failures and hypertension, as long as inhibiting the β-adrenergic receptors, exhibits an antioxidant effect that can lead to increased drug efficacy in the treatment of the cardiac failure (40, 47). Subcutaneous injection of isoproterenol, a beta-adrenergic receptor agonist, triggers myocardial hyperactivity and instant increased heart rate (48-50).

Myocardial infarction initiates the heart rate increment and reduction of perfusion to the organs. Subsequently, the adrenergic system is activated to compensate the systemic hemorrhage. Heart remodeling, necrosis and heart tissue damages occur as a result of the adrenergic system activation. CAR, as a β blocker, prevents activation of the adrenergic system, reduces infarct size, decreases histological damage, regulates the heart rate and inhibits the later consequences of necrosis and heart remodeling. Additionally, the antioxidant activity of CAR neutralizes the reactive oxygen species (ROS) caused by infarction (40, 51, 52). Numerous studies have been conducted to evaluate the effects of various processing parameters and find an appropriate way to improve the *in vitro* and *in vivo* characteristics of CAR. Venishetty *et al.* prepared N-carboxymethyl chitosan coated CAR-loaded solid lipid nanoparticles to increase the oral bioavailability of the drug in the rats (53). In another study, CAR-loaded mucoadhesive chitosan microspheres were developed and efficaciously administered nasally in the rabbits (54). To come to the point, our findings are in good agreement with the previously published works regarding clinical (39, 40) and animal studies (3, 47, 53, 54) of the different formulations of CAR. Therefore, CAR-Eud electrosprayed nanostructures could be interestingly considered as feasible candidates for enhancement of the drug therapeutic effectiveness.

## Conclusion

Nanobeads and nanofibers of CAR (as a poorly water soluble drug) were effectively formulated using electrospraying technique. The microstructure studies revealed that the drug: polymer ratios (1:5 and 1:10) as well as the total solution concentrations variation 0-20 % (w/v) particularly affected the size and morphology of the nanostructures, where by increasing the solution concentration the beads size increased and the highest concentration led to the fiber formation. DSC and PXRD results showed that the crystalline structure of CAR was transformed to the amorphous form during the electrospraying procedure. Based on the *in vitro* drug release tests, the electrosprayed nanosystems depicted meaningfully faster drug release rate than the PMs and pure drug. This could be attributed to the amorphization of CAR, homogenous dispersion of the drug into the polymeric matrix and its enhanced specific surface area due to the smaller size of the prepared nanosystems. Furthermore, according to the *in vivo* results, the electrosprayed nanoformulations reduced the ISO induced elevation of the heart rate and reduced ISO induced necrosis and accumulation of neutrophils in the heart tissue more effective than the pure drug and PM. Our finding revealed that the electrospraying as an economic and one step technique could be effectively applied for improving the physicochemical characteristics and pharmacologic effect of CAR.
